# Chikungunya Virus Detection in *Aedes aegypti* and *Culex quinquefasciatus* during an Outbreak in the Amazon Region

**DOI:** 10.3390/v12080853

**Published:** 2020-08-04

**Authors:** Ana Cecília Ribeiro Cruz, Joaquim Pinto Nunes Neto, Sandro Patroca da Silva, Eliana Vieira Pinto da Silva, Glennda Juscely Galvão Pereira, Maissa Maia Santos, Hamilton Antônio de Oliveira Monteiro, Flavia Barreto dos Santos, Ricardo José de Paula Souza e Guimarães, Carine Fortes Aragão, Lívia Carício Martins

**Affiliations:** 1 Seção de Arbovirologia e Febres Hemorrágicas, Instituto Evandro Chagas, Secretaria de Vigilância e Saúde, Ministério da Saúde, Ananindeua, PA 67030-000, Brazil; anacecilia@iec.gov.br (A.C.R.C.); spatroca@gmail.com (S.P.d.S.); elianapinto@iec.gov.br (E.V.P.d.S.); bioagl2013@gmail.com (G.J.G.P.); maissasantos@iec.gov.br (M.M.S.); hamiltonmonteiro@iec.gov.br (H.A.d.O.M.); ricardoguimaraes@iec.gov.br (R.J.d.P.S.eG.); carinefaragao@gmail.com (C.F.A.); liviamartins@iec.gov.br (L.C.M.); 2 Departamento de Patologia, Universidade do Estado do Pará, Belém, PA 66087-662, Brazil; 3 Programa de Pós-Graduação em Virologia, Instituto Evandro Chagas, Ananindeua, PA 67030-000, Brazil; 4 Laboratório de Imunologia Viral, Fundação do Instituto Oswaldo Cruz, Rio de Janeiro, RJ 21040-900, Brazil; flaviab@ioc.fiocruz.br

**Keywords:** Chikungunya, *Aedes aegypti*, *Culex quinquefasciatus*, East-Central-South-Africa genotype, entomological surveillance, Amazon, Brazil

## Abstract

Chikungunya virus (CHIKV) was first reported in Brazil in 2014 and, after it spread countrywide, an outbreak of febrile illness with reports of arthralgia happened in the municipality of Xinguara, Pará, Brazil in 2017, indicating the virus’ circulation. Here, we aimed to investigate CHIKV in mosquito vectors collected during an active surveillance of virus isolation in cell culture by using molecular detection and viral genome sequencing. A total of 492 *Aedes*, *Culex* and *Mansonia* mosquitoes were collected and separated in 36 pools according to the species and sex, and 22.2% (8/36) were positive. CHIKV was indentified in pools of *Ae. aegypti* females (*n* = 5), an *Ae. aegypti* male (*n* = 1) and in *Culex quinquefasciatus* females (*n* = 2). However, as the mosquitoes’ whole bodies were macerated and used for detection, one cannot suggest the role of the latter in the viral transmission. Despite this, vector competence studies must be carried out in the different species to investigate long-term adaptations. Viral genome sequencing has characterized the East-Central-South-African (ECSA) genotype in all positive pools analyzed, corroborating previous reports for the Amazon region.

## 1. Introduction

Chikungunya virus (CHIKV) is a single-strand RNA virus belonging to the *Togaviridae* family, of the *Alphavirus* genus, transmitted by mosquitoes and often causing an acute febrile illness accompanied by severe and debilitating arthralgia in human-chikungunya fever [1]. CHIKV caused massive and sustained outbreaks in Asia and Africa between the 1960s and 1980s. After this period, there was a decrease in the number of cases, until 2004, when an epidemic was reported in Kenya, Africa, resulting in the occurrence of outbreaks in both the aforementioned continents [2,3]. In the Americas, only imported cases from endemic Asian and African areas were reported for almost a decade [3], however, in 2013, the first autochthonous cases were confirmed in the Caribbean islands [4]. In the following year, Brazil confirmed the first autochthonous cases in Oiapoque, state of Amapá, the North region, and Feira de Santana, of Bahia, the Northeast region [5] and soon, the virus spread nationwide in the following years, resulting in epidemics in 2016 and 2017 [6,7].

Phylogenetic studies revealed the worldwide circulation of four CHIKV genotypes, the names of which were based on their geographical distribution: (I) West Africa, (II) East-Central-South Africa (ECSA), (III) Asia and (IV) Indian Ocean (IOL) [8]. The Asian genotype was responsible for the first cases of chikungunya in the Americas [2] and, in Brazil, it was introduced in the state of Amapá, while, in Bahia, the first infections were caused by the ECSA genotype [9]. Currently, the two genotypes remain co-circulating in the country and are the only ones reported so far.

The rapid spread and establishment of CHIKV in Brazil was facilitated by the high density of its main vector—*Aedes (Ae.) aegypti*, a species of great importance in the viral transmission dynamics of the country [10]. Moreover, the favorable climate, associated with the disordered urbanization and wide availability of reservoirs serving as breeding sites, favored the proliferation of these vectors and, consequently, increased the viral transmission [11,12,13]. Therefore, mosquito control is still the main strategy to prevent epidemics. The lack of a specific treatment and vaccine reinforces the importance of vector control [14]. After zika emergence, vector control strategies were intensified, but were not enough to avoid the increased number of *Ae. aegypti*-related diseases [15].

According to the Brazilian Ministry of Health (MoH), in 2016 and 2017 a total of 271,824 and 185,593 cases of chikungunya were confirmed in Brazil, with 196 and 173 deaths, respectively [6,7]. In the North region, the state of Pará had the highest number of notifications, reporting 48.2% (4343/9019) and 51.3% (8505/16,570) of the cases from the North region, respectively [7]. In 2017, the municipality of Xinguara, Pará, reported an outbreak of febrile illness associated with severe arthralgia, suggesting the occurrence of chikungunya infections. Therefore, we aimed to investigate the CHIKV infection in mosquitoes collected in the urban areas of Xinguara, located in the Amazon region of Brazil.

## 2. Materials and Methods 

### 2.1. Study Area

**Figure 1:**
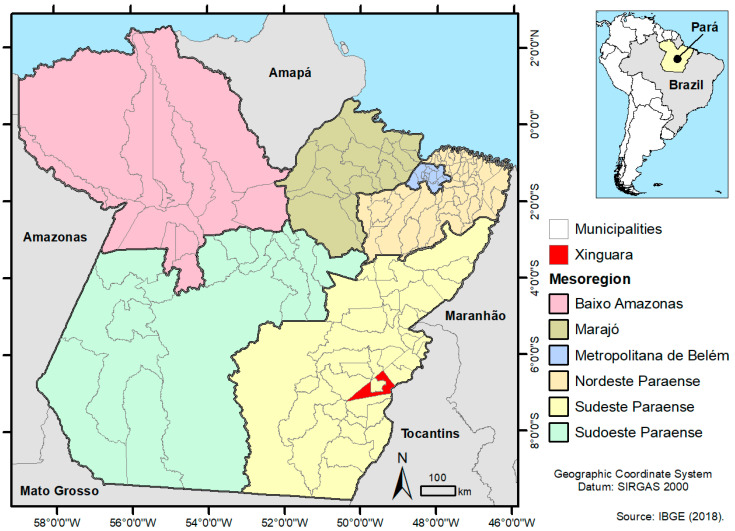
Study area. Municipality of Xinguara, located in the Southeast Mesoregion of the state of Pará, North Brazil.

This study was carried out in the municipality of Xinguara, located in the Southeast Mesoregion of the state of Pará, North Brazil (Figure 1). The municipality has 40,573 inhabitants and an area of 3779.348 km^2^, is located in the Amazon and, therefore, suffers the environmental influences typical of the region [16]. The vegetation is represented by the equatorial latifoliated forest, presenting areas of mixed and cerrado forest. The relative humidity is high (an average of 78%), ranging from 90% in the rainy season (November to May) to 52% in the dry season (June to October), and an average annual temperature of 26.35 °C and a rainfall of around 2000 mm [17].

### 2.2. Mosquitoes Collection and Identification

The mosquitoes were collected by a team from the Arbovirology and Hemorrhagic Fevers Section (SAARB) of the Medical Entomology Laboratory and the Evandro Chagas Institute (IEC), as requested by the MoH to investigate suspected chikungunya cases in Xinguara in January of 2017. The active surveillance was performed using a puçá and consisted of capturing the mosquitoes in flight, which were then retained in a fabric net. The collection was performed inside residences located in: the Centro, Itamarati, Tanaka II and Novo Horizonte neighborhoods; inside and outside residences in the Frei Henry neighborhood, and near the building of an Emergency Care Unit (ECU), located in the Centro neighborhood (Figure 1). The collected mosquitoes were immediately transferred, with the aid of an oral suction device, to tubes with the appropriate date and place of collection, stored in liquid nitrogen at −196 °C and sent to the IEC for species identification using dichotomous keys [18,19,20,21,22,23,24] in a refrigerated table at −20 °C. Once identified, the mosquitoes were quantified and grouped in pools, according to species, sex and place of collection. The pools were identified with an exclusive registration, adopted by SAARB/IEC, composed by the acronym AR (of ARtropod) followed by a sequential number.

### 2.3. Mosquitoes Preparation

The mosquitoes’ suspensions were obtained by macerating each pool in 1 mL of a solution composed of Dulbecco’s Phosphate Buffered Saline 1X (D-PBS (Life technologies, Carlsbad, CA, USA)), 2% penicillin and streptomycin, 1% fungizone and 5% fetal bovine serum (FBS). Macerates were obtained using a TissueLyser II (Qiagen, Hilden, Germany) at a frequency of 25 Hz for 1 min, and with the aid of a 3 mm tungsten sphere, based on an adapted protocol [25]. The macerates were kept at −70 °C for 12 h.

### 2.4. Virus Isolation

As the gold standard test for investigating arboviruses, the mosquitoes’ macerates were submitted to virus isolation using *Aedes albopictus* C6/36 clone (ATCC: CRL1 660) cells seeded in 10 mL culture tubes containing 1.5 mL of glutamine-modified Leibowitz medium (L-15), with 2.95% tryptose phosphate, non-essential amino acids and antibiotics (penicillin and streptomycin), and 2% fetal bovine serum, for a period of three days. Prior to inoculation, the mosquito pools’ macerates were centrifuged for 10 min, at 10,000× *g* rpm, at 4 °C. The cell culture medium was discarded and 100 µL of the sample supernatant was inoculated into the cell monolayers and incubated at 28 °C for one hour for adsorption, with stirring performed every 15 min. Inoculated cells were kept in the 1.5 mL L-15 medium, with 2.95% tryptose phosphate, non-essential amino acids, antibiotics (penicillin and streptomycin) and 2% FBS, according to an adapted protocol [26]. Inoculated cells were kept at 28 °C and were observed daily for a period of 7 days or until the presence of a cytopathic effect (CPE). 

### 2.5. Indirect Immunofluorescence Test 

Virus identification was performed by using immunofluorescence, as previously described [27], with modifications. Briefly, the inoculated cells were harvested, fixed for ten minutes in acetone and kept at −20 °C. Subsequently, 10 μL of polyclonal antibodies was added to arboviruses of the *Alphavirus*, *Flavivirus*, *Orthobunyavirus* and *Phlebovirus* genuses, diluted to 1:20 in PBS with a pH of 7.4 and incubated for 30 min. After an incubation at 37 °C, with 5% CO_2_, cells were washed three times in PBS with a pH of 7.4 and were given a quick wash in distilled water. After air drying, 10 μL of anti-mouse conjugate (Cappel) diluted to 1:900 was added and the slides were incubated for 30 min. Next, slides were analyzed in a fluorescence microscope (Olympus BX51, UPlanFL N 20X/0.5 objective and WB and U-25nd filters, Olympus, Tokyo, Japan). Positive samples, identified by their immunofluorescence, were further tested by the RT-qPCR for confirmation and subsequent nucleotide sequencing.

### 2.6. Viral RNA Extraction and Real-Time Reverse Transcriptase Polymerase Chain Reaction (RT-qPCR)

Viral RNA extraction was performed using the QIAamp^®^ viral RNA kit (Qiagen, Hilden, Germany), according to the manufacturer’s instructions. A non-competitive internal control (genomic phage RNA MS2, Roche Diagnostics, Basel, Switzerland) [28] was used as positive control and as a negative control, the latter of which consisted of nuclease-free water. The RT-qPCR assay was performed using the SuperScript^®^III Platinum^®^One-Step RT-qPCR Kit (ThermoFisher Scientific, Waltham, MA, USA), which contains the specific primers and probe for detecting the CHIKV NSP1 protein [29]. Briefly, the 25 μL reaction consisted of 12.5 μL of reaction mix (2× concentrated), 5.5 μL of nuclease-free water, 1.0 μL of Forward and Reverse primer, 0.5 μL of probe, 0.5 μL of the Super Script III platinum Taq Mix enzyme and 5 μL of the extracted RNA, and was performed on the 7500 Fast Real-Time PCR system (ThermoFisher Scientific, Waltham, MA, USA). The cycling conditions consisted of an initial RT step at 50 °C for 30 min, followed by a 2 min denaturation step at 95 °C, and then 45 cycles of 15 s at 95 °C and 1 min at 60 °C. Positive (CHIKV infected mouse brain) and negative (nuclease-free water) controls were included. The samples were analyzed in duplicate and considered as positive when the average cycle threshold (Ct) value was less than 37.

### 2.7. Genome Sequencing and Phylogenetic Analysis

The viral genome was recovered by synthesizing the first and second complementary DNA (cDNA) strands, constructed directly from the single-stranded RNA (ssRNA). Briefly, the synthesis was performed using the cDNA Synthesis System kit (Roche Diagnostics, Basel, Switzerland) and 400 µM of Roche random primer. The products were purified using magnetic beads (Agencourt AMPure XP Reagent, Beckman Coulter, CA, USA) and were given three washes with 800 µL of 70% ethanol. The cDNA was eluted in 16 µL of 10 mM of Tris-HCl (pH 7.5). The cDNA library was prepared and sequenced using the protocol described in the Nextera XT DNA Library Preparation Kit on a MiniSeq platform (Illumina Inc., San Diego, CA, USA).

The genome assembly was carried out using the De Novo Assembler methodology in the IDBA-UD program [30]. All the contigs were aligned and compared to the virus protein (RefSeq) database available in National Center for Biotechnology Information (NCBI) through DIAMOND [31]. The result was visualized using Megan6 software [32] and the inspection and annotations of putative open reading frame (ORF) genes were performed using Geneious v. 9.1.6 software (Biomatters, Auckland, New Zealand).

A multiple sequence alignment (MSA) was performed using the Mat v. 7 program [33]. Before phylogenetic analysis, the ProtTest was applied to select the best-fit models of amino acid substitution [34]. The reconstruction of phylogenetic trees was performed using the maximum likelihood (ML) method [35] implemented in RaxML v. 8.2.4 [36]. To determine the reliability of the tree topology, a bootstrap analysis [37] was carried out on 1000 replicates.

## 3. Results

### 3.1. Collection and Identification

A total of 492 mosquitoes were collected and 36 pools were formed. *Cx. quinquefasciatus* (68.7%, 47.6% females and 21.1% males) and *Ae. aegypti* (28.6%, 21.3% females and 7.3% males) were the predominant species collected. *Ae. albopictus* specimens were collected in the Centro and Frei Henry neighborhoods. One *Ae. scapularis* female and one *Ae. serratus* female were also collected in Centro. Among the collection sites, the largest number of specimens (55.1%; 319/492) were collected in the Centro neighborhood, where most *Ae. aegypti and Cx. quinquefasciatus* specimens, as well as the only *Mansonia* (*Mansonia*) specie were collected (Table 1).

### 3.2. Virus Isolation and Molecular Detection

All pools were submitted to virus isolation and 22.2% (8/36) were positive. CHIKV was isolated in pools of *Ae. aegypti* females (*n* = 5), *Ae. aegypti* male (*n* = 1) and in *Cx. quinquefasciatus* females (*n* = 2). After RT-qPCR, a CHIKV infection was confirmed in seven pools, as one pool was no longer available after virus isolation for molecular detection. RT-qPCR Ct values observed on the CHIKV positive pools of *Ae. aegypti* females ranged from 20.2 to 23.3, and after isolation in cell culture, Ct values ranged from 12.1 to 13.9. The two CHIKV positive pools of *Cx. quinquefasciatus* females presented Ct values of 28.2 and 33.7, and after isolation in cell culture, those were 13.4 and 18.9, respectively (Table 2).

### 3.3. Phylogenetic Analysis

**Figure 2:**
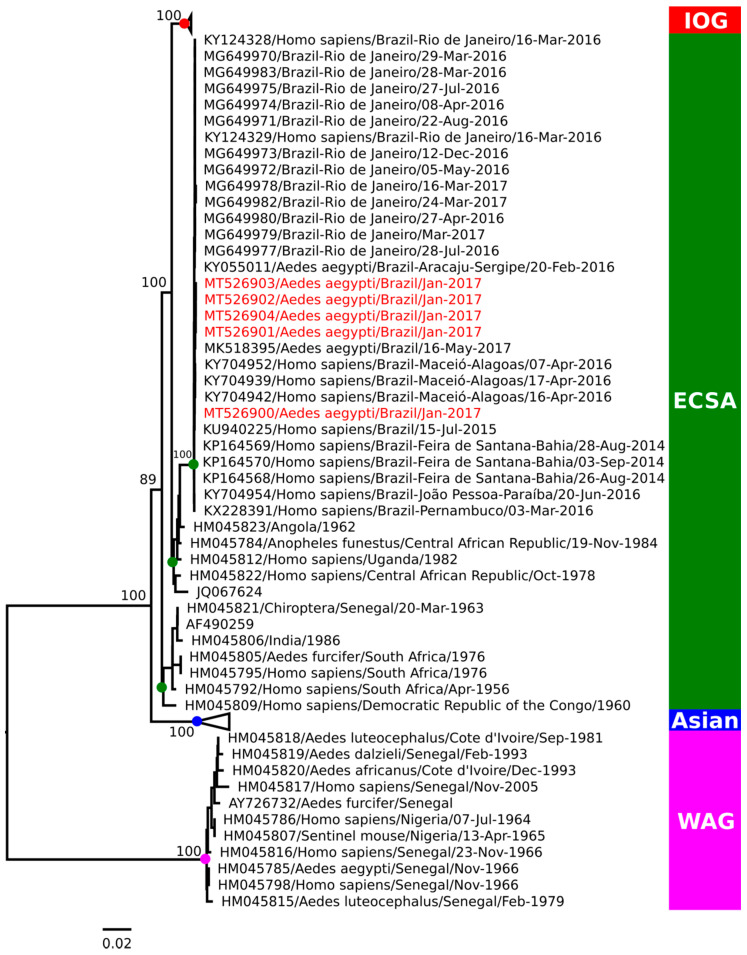
Maximum likelihood phylogenetic analysis based on the complete genome of chikungunya virus (CHIKV) isolated in females of *Ae. aegypti* and *Cx. quinquefasciatus*, collected during an outbreak in the Amazon region in 2017. The strains sequenced in this study are in red. Colored circles—red, green, blue and magenta—on the tree nodes represent the Indian Ocean, ECSA, Asian and West Africa clusters, respectively.

The CHIKV positive pools AR843521 (GenBank access number MT526900) and AR843523 (access number MT526901) are representative of *Ae. aegypti* females; AR843528 (access number MT526902) and AR843529 (access number MT526903) are representative of the *Cx. quinquefasciatus* females, all collected on the Centro site; AR843544 (access number MT526904) is representative of the *Ae. aegypti* females collected on the Tanaka II site, were all selected for viral genome sequencing. Phylogeny based on the complete genome analysis characterized the CHIKV, in all the pools analyzed, as belonging to the ECSA genotype (Figure 2), with a high similarity to other samples previously detected in North and Northeast Brazil.

## 4. Discussion

In the present study, we identified CHIKV in *Ae. aegypti* (males and females) and *Cx. quinquefasciatus* (females), confirming that virus circulation took place during an outbreak in the municipality of Xinguara, Pará in 2017. *Ae. aegypti* mosquitoes are a worldwide recognized vector for CHIKV and, in Brazil, female mosquitoes of the same species have already been found naturally infected in the states of Sergipe [38] and Maranhão [39,40], both in the Northeast region of the country. A recent study in an urban park in Rio Grande do Norte, also in the Northeast, identified CHIKV in pools of *Ae. aegypti* females, *Ae. albopictus* females and males, in *Ae. fluviatilis* and in *Wyeomyia bourrouli* [41]. However, this is the first report of an *Ae. aegypti* male naturally infected by CHIKV in the country, which implies transovarian transmission. Populations of *Ae. aegypti* males collected in Mexico [42] and Thailand [43] have also been identified to be naturally infected with CHIKV. It has been shown that the high vector competence of both *Ae. aegypti* and *Ae. albopictus* in transmitting CHIKV plays an important role in the virus spread in American countries [44].

We also reported the natural infection of *Cx. quinquefasciatus*, however, one can not speculate and imply that the species is participating in the CHIKV transmission. One limitation of this study is that the mosquitoes had their bodies macerated and analyzed as a whole, and salivary gland analysis, that could be indicative of vector competence, was not performed.

Most mosquito pools that tested positive for CHIKV were collected in the Centro neighborhood near an ECU, which is of particular concern, as it constitutes a highly busy area, with an intense population movement seeking medical assistance and which may have favored the virus dissemination. Therefore, it is suggested that vector control measures shall be intensified in such areas.

In this study, phylogeny characterized the CHIKV ECSA genotype in all the pools analyzed. The sequence recovered from the sample AR843521 was closely related to those previously collected from Bahia in 2015 (GeneBank accession number KU940225) [45] and Alagoas in 2016 (KY704942) [46], while the others (AR843523, AR843528, AR843529 and AR843544), were more related to viruses identified in *Ae. aegypti* collected in Sergipe in 2016 (KY055011) [38] and Maranhão in 2017 (MK518395) [40]. The same genotype has already been reported during an outbreak in the state of Roraima [47], also in the North region of Brazil, demonstrating this genotype’s dissemination and persistence in the region.

The CHIKV ECSA genotype was first reported in Brazil in 2014, in Feira de Santana, Bahia, in the Northeast region and, in the following years, spread to other states, being identified in Bahia (2014–2017) [9,48,49], Alagoas (2016) [46,50], Piauí (2016–2017) [51], Sergipe (2016) [38], and Maranhão (2017) [40]. It was also identified in the Southeast region, in Rio de Janeiro (2016–2018) [52,53] and Minas Gerais (2017) [54]. Moreover, it has been suggested that the probable introduction of the ECSA genotype in the North occurred from the Northeast in mid-2015 [55]. However, the circulation of the ECSA genotype in the North may be troublesome, as the Asian genotype already circulated in the region since its first introduction in the country, in Oiapoque, Amapá [9]. Surprisingly, their co-infection has already been reported in a recent study in human individuals naturally infected in the Northeast region [55]. It has been shown that both the Asian and ECSA genotypes could spread and co-circulate in the country, considering the vector species suitability [9].

Despite the reports indicating that the introduction of the ECSA genotype was in Brazil in 2014, a recent study showed that this genotype has been circulating in Brazil since 2013, almost a year before it was detected in humans [56]. This highlights the role of entomological surveillance, as the virus may be circulating silently, before human infections may be detected. Furthermore, in recent years, entomo-virological surveillance has proven to be a useful tool for detecting viruses of medical importance during outbreaks, monitoring virus circulation and characterizing vectors, which are crucial in understanding the dynamics of viral transmission.

In this study, the entomological surveillance complemented the clinical observation on the occurrence of chikungunya in Xinguara, Pará, and presented data that reinforce the need and importance of vector competence studies in different species of mosquitoes. The vector and virological data, associated with human diagnosis, are imperative to establish strategies for the disease control.

## Figures and Tables

**Table 1:** Distribution of mosquitoes according to species, sex and the collection site in Xinguara, Pará during an outbreak of chikungunya, 2017.

Species (♀ or ♂)	Collection Sites										Total		
	Centro		Itamarati		Tanaka II		Frei Henry		Novo Horizonte				
	N ^1^	P ^2^	N	P	N	P	N	P	N	P	N	P	%
*Ae. aegypti ♀*	84	5	5	1	4	1	5	1	7	1	105	9	21.3
*Ae. aegypti ♂*	28	2	3	1	4	1	0	0	1	1	36	5	7.3
*Ae. albopictus ♀*	2	1	0	0	0	0	5	1	0	0	7	2	1.4
*Ae. albopictus ♂*	0	0	0	0	0	0	3	1	0	0	3	1	0.6
*Ae. scapularis ♀*	1	1	0	0	0	0	0	0	0	0	1	1	0.2
*Ae. serratus ♀*	1	1	0	0	0	0	0	0	0	0	1	1	0.2
*Cx. quinquefasciatus ♀*	152	6	11	1	34	1	29	1	8	1	234	10	47.6
*Cx. quinquefasciatus ♂*	50	2	6	1	36	1	10	1	2	1	104	6	21.1
*Mansonia (Mansonia) sp. ^3^ ♀*	1	1	0	0	0	0	0	0	0	0	1	1	0.2
Total	**319**	**19**	**25**	**4**	**78**	**4**	**52**	**5**	**18**	**4**	**492**	**36**	**100**

^1^ Number of mosquitoes collected; ^2^ Number of pools; ^3^ Species.

**Table 2:** Investigation of mosquito pools by virus isolation and RT-qPCR during the chikungunya outbreak in Xinguara, Pará, 2017.

Pool Number	N. ^1^	Species (♀ or ♂)	Collection Date	Collection Site	Virus Isolation	RT-qPCR			
						Pool	Ct ^2^	Cell Supernatant	Ct ^2^
AR843521	15	*Ae. Aegypti* *♀*	24 January 2017	Centro	CHIKV ^3^	CHIKV	21.3	CHIKV	13.9
AR843522	21	*Ae. aegypti* *♀*	25 January 2017	Centro	CHIKV	CHIKV	21.3	CHIKV	12.1
AR843523	21	*Ae. aegypti* *♀*	25 January 2017	Centro	CHIKV	CHIKV	23.3	CHIKV	13
AR843524	23	*Ae. aegypti* *♀*	26 January 2017	Centro	CHIKV	CHIKV	21	CHIKV	13.9
AR843525	26	*Ae. aegypti* *♂*	24 January 25	Centro	CHIKV	ND ^4^	-	ND ^4^	-
AR843528	35	*Cx. quinquefasciatus* *♀*	25 January 2017	Centro	CHIKV	CHIKV	28.2	CHIKV	13.4
AR843529	27	*Cx. quinquefasciatus* *♀*	26 January 2017	Centro	CHIKV	CHIKV	33.7	CHIKV	18.9
AR843544	4	*Ae. aegypti* *♀*	25 January and	Tanaka II	CHIKV	CHIKV	20.2	CHIKV	12.5

^1^ Number of mosquitoes collected; ^2^ Cycle threshold value; ^3^ *Chikungunya virus*; ^4^ Not done; ♀: Female; ♂: Male.
